# How necessary is a fast testkit for mitigation of pandemic flu?

**DOI:** 10.1098/rsif.2009.0471

**Published:** 2009-12-18

**Authors:** Juxin Chin, Geoffrey Koh, Dong-Yup Lee

**Affiliations:** 1 Bioprocessing Technology Institute, Agency for Science, Technology and Research (A*STAR), 20 Biopolis Way, #06-01, Centros, Singapore 138668, Republic of Singapore; 2 Department of Chemical and Biomolecular Engineering, National University of Singapore, 4 Engineering Drive, Singapore 117576, Republic of Singapore

**Keywords:** intervention policy, stochastic agent-based model, fast testkit, simulation, pandemic outbreak

## Abstract

It is widely feared that a novel, highly pathogenic, human transmissible influenza virus may evolve that could cause the next global pandemic. Mitigating the spread of such an influenza pandemic would require not only the timely administration of antiviral drugs to those infected, but also the implementation of suitable intervention policies for stunting the spread of the virus. Towards this end, mathematical modelling and simulation studies are crucial as they allow us to evaluate the predicted effectiveness of the various intervention policies before enforcing them. Diagnosis plays a vital role in the overall pandemic management framework by detecting and distinguishing the pathogenic strain from the less threatening seasonal strains and other influenza-like illnesses. This allows treatment and intervention to be deployed effectively, given limited antiviral supplies and other resources. However, the time required to design a fast and accurate testkit for novel strains may limit the role of diagnosis. Herein, we aim to investigate the cost and effectiveness of different diagnostic methods using a stochastic agent-based city-scale model, and then address the issue of whether conventional testing approaches, when used with appropriate intervention policies, can be as effective as fast testkits in containing a pandemic outbreak. We found that for mitigation purposes, fast and accurate testkits are not necessary as long as sufficient medication is given, and are generally recommended only when used with extensive contact tracing and prophylaxis. Additionally, in the event of insufficient medication and fast testkits, the use of slower, conventional testkits together with proper isolation policies while waiting for the diagnostic results can be an equally effective substitute.

## 1. Introduction

A global influenza pandemic has the potential to cause extensive morbidity and mortality, as well as severe social and economic disruptions. Past and present pandemics such as the Asian flu (1957, H2N2), the Hong Kong flu (1968, H3N2) and the recent swine flu (2009, H1N1) outbreaks have demonstrated the extent to which the virus can be transmitted swiftly on a global scale (Cox & Subbarao 2000; Fraser *et al*. 2009). In the case of the 2009 H1N1 flu pandemic, although the H1N1 strain is not as lethal as originally thought, there are fears that a deadlier strain may emerge, sparking off the next wave of health crisis. This is in addition to the existing threat of the highly lethal H5N1 avian influenza virus (Hatta *et al*. 2001) mutating into a human-transmissible variant (Hatta & Kawaoka 2002).

It is recognized that a combination of early use of antiviral medicine and social distancing measures can help contain a pandemic, or at least slow its spread until proper vaccines can be developed (Nuño *et al*. 2007). However, due to limited medicine stockpiles and resources, policies must be made such that they optimize the drug usage while minimizing the cost and economic impact of the intervention strategies. Since it is not possible to experimentally assess the overall effectiveness of the various pandemic control strategies, mathematical models play a major role as they allow us to address such issues through simulations. Notable past works include examining how to contain a novel influenza strain at its source (Ferguson *et al*. 2005; Longini *et al*. 2005), and failing that, how we can limit its impact within the population (Ferguson *et al*. 2006; Germann *et al*. 2006; Wu *et al*. 2006; Nuño *et al*. 2007).

Diagnosis is an important component in the overall pandemic management framework. With proper diagnosis, potential flu carriers can be identified before they become symptomatic. Non-pharmaceutical measures such as quarantine and contact tracing can then be activated, thus maximizing the probability of containment (Ferguson *et al*. 2005; Longini *et al*. 2005). Even in the event of an ongoing outbreak, it was found that early detection and intervention can help mitigate the spread of influenza. For instance, studies have revealed that during the 1918 flu pandemic, cities in the United States that enforced early interventions had significantly reduced mortality rate (Hatchett *et al*. 2007). Furthermore, during a pandemic, various influenza-like illnesses (ILIs), as well as non-pandemic seasonal influenza cases will continue to present themselves within the population. The ability to differentiate between the pathogenic strains and the less harmful ones can help channel antiviral drugs to where they are needed. Such targeted treatment is invaluable in the face of limited drug supplies, especially in developing countries.

Current influenza diagnostic tests, especially when the strains are evolving constantly, vary differently in terms of efficiency, specificity and sensitivity (for a list of diagnostic tests, see Centers for Disease Control and Prevention 2009). These factors will impact the intervention policies that should be made during an outbreak. It has been argued that a slow, low throughput laboratory-based diagnostic test, such as immunofluorescence DFA antibody staining and RT-PCR (real-time polymerase chain reaction), may not be able to effectively assist pandemic mitigation (Wu *et al*. 2006). Ideally, a rapid PCR-based diagnostic testkit should be able to detect the relevant virus in less than 30 minutes, requiring nothing more than a patient sample, and a portable device to perform extraction and drive the PCR cycle. This permits testing to be done quickly, outside a laboratory setting, and allows diagnosis to be performed more easily in rural areas, where novel influenza is likely to emerge. However, such testkits require time to develop for novel strains, and may not be available during the initial phases of a pandemic, during which one may have to fall back on conventional laboratory-based approaches.

The constraints imposed by the availability of fast and accurate diagnostic methods are factors that should not be overlooked in flu intervention policy making. However, previous works on pandemic modelling either treat ILIs as false positives that consume resources, or assume perfect diagnosis without accounting for the costs associated with the use of the different diagnostic techniques. In this work, we aim to investigate the cost and effectiveness of various intervention policies when implemented with different diagnostic methods. Specifically, we want to address the following questions—Is a fast and accurate testkit necessary in mitigating the spread of an ongoing pandemic? In addition, are there any other dominating factors that should be given due consideration in forming pandemic mitigation policies? To do so, we developed a stochastic agent-based epidemic framework and implemented it on a small city-scale model, typified by developed countries such as Singapore and the United States. The impact of testkits when used in the context of different intervention policies is then assessed by comparing the severity of the outbreak with an aggregate cost function which encompasses the various costs and resources involved.

## 2. Methods

### 2.1. Model outline

**Figure 1.**
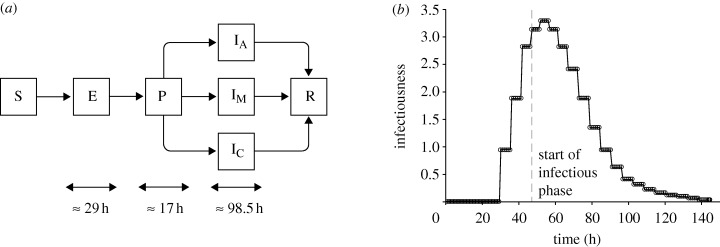
Transmission model and infectiousness profile. (*a*) The state transition diagram depicting the SEPIR (susceptible–exposed–pre-symptomatic–infectious–recovered) transmission model for pandemic influenza. The infectious phase is further sub-divided into asymptomatic (I_A_), mild (I_M_) and critical (I_C_) cases. (*b*) The mean relative infectiousness of a person throughout the various phases.

For our work, we developed a stochastic agent-based pandemic model on a small-scale city. We extended the typical transmission model (Wu *et al*. 2006) by adding a pre-symptomatic phase, thus resulting in the SEPIR (susceptible–exposed–pre-symptomatic–infectious–recovered) model. In addition, we further refined the infectious phase into one of the three possible categories—asymptomatic, symptomatic and critical (for details, refer to figure 1*a*). To capture the constraints imposed by societal norms, such as the different population response patterns during different times of the day, we set the granularity of the time scale to be in terms of hours, rather than days.

We populated the city with 10 000 people, and simulation proceeds in discrete time steps of 1 h. Within the city, we identified the following location types—(i) household, (ii) workplace, (iii) school, (iv) mall, (v) hospital, and (vi) public transport. Whenever the context is clear, both workplaces and schools are simply referred to as workplaces, while malls, hospitals and public transport are termed communities. For workplaces, schools and malls, they are further divided into sub-locations. Workplaces have smaller work groups, schools are divided into classes, and malls are made up of several shops. At any point in time, each individual in the city will be in any one of the locations or sub-locations. Note that we classified hospital as a separate location entity, as it will be the main facility where diagnosis and treatment can be issued. The number of people in each location was obtained by fitting the model against demographic data for Singapore (Statistics Singapore 2009).

Each location in the model, with the exception of public transport, has an *x*–*y* coordinate assigned to it on a 10 × 10 square grid. Inhabitants of the city move from one location to the next according to their individual schedules. Commuting between grids is done by either private (i.e. cars) or public transport. However, between locations on the same grid coordinates, it is assumed that movement does not require any forms of transport. Public transport is considered as a location. Instead of *x*–*y* coordinates, it is assigned to a route it serves. The other locations in the city are grouped into four districts (each occupying a quadrant in the city grid). Travelling within and between the districts is done via a specific transport route, giving a total of 16 routes. All individuals travelling along the same route are considered to be in the same location, thus allowing the spread of pandemic flu.

In the city, we assumed that there are two hospitals, with one being designated as a flu treatment and isolation centre during a pandemic outbreak. Patients with flu-like symptoms will be directed to that hospital for diagnosis and treatment. The other hospital will handle other non-influenza-like illnesses (ONILIs) which include cases such as bacterial infections and accidental injuries. Normally, without a designated flu hospital, these ONILI cases will present themselves as susceptible targets for infection within the hospital. However, this scenario is not considered in depth within our model.

As mentioned in the previous section, ILI is one of the sources of drug wastage. It represents other types of illnesses as well as non-pandemic seasonal influenza, and is symptomatically indistinguishable from the pandemic strain. For these patients, they will be directed to the designated flu centre for diagnosis and treatment. Without proper diagnoses, these patients will consume resources unnecessarily. Since ILIs are non-specific illnesses, we assumed the number of new ILI cases to surface at a constant rate, independent from the spread of pandemic influenza. Variations due to seasonal effects are currently beyond the scope of this work. Based on published data from the Ministry of Health, Singapore (Ministry of Health Singapore 2006*b*), we estimated the rate to be three new infections per day, each lasting for 72 h. In the model, these individuals are randomly selected from the ILI-susceptible population; those suffering from pandemic flu and ONILIs can still be inflicted by ILIs. For ONILIs, we estimated the daily new occurrences to be 45 people (Ministry of Health Singapore 2006*a*), and they are randomly selected from the ONILI-susceptible population. Again, each ONILI case is assumed to last for 72 h. For details on how the values are derived, refer to the electronic supplementary material.

### 2.2. Natural history of influenza

To model the spread of novel pandemic virus, parameters governing its infection dynamics are required. Since such parameters are not readily available for novel variants, we referred to the natural history of typical influenza viruses, adapting the values from previous works whenever possible. The basic function describing the dynamics of transmission is the instantaneous force of infection experienced by the *i*th individual, and it is summarized by the following equation:
2.1
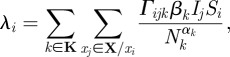

where **X** and **K** are the sets of people and locations in the city, respectively; *β*_*k*_ is the transmission coefficient associated with a particular location type. The transmission coefficients are estimated such that without any forms of intervention, the proportions of transmissions within the household (*P*_H_), workplace (*P*_W_) and community (*P*_C_) are approximately 30, 37 and 33 per cent, respectively (Ferguson *et al*. 2006). This proportion is location-dependent, and the values we followed are reported to be estimated based on data from the United States. Using our transmission coefficients (*β*_h_ = 1.89 × 10^−1^ h^−1^, *β*_w_ = 3.2 × 10^−1^ h^−1^ and *β*_c_ = 6.51 × 10^−3^ h^−1^) the transmission proportions in the model are computed to be *P*_H_ = 30.7 per cent, *P*_W_ = 35.6 per cent and *P*_C_ = 33.7 per cent. *Γ*_*ijk*_ is a proximity function for two individuals *x_i_*, *x_j_* and location *k* at a particular time instance. In the model, the function will give 3 if *x_i_* and *x_j_* are in the same sub-location and 1 if they are in the same location, but different sub-locations. Otherwise it returns 0. *I_j_* and *S_i_* are parameters that describe the infectiousness of *x_j_* and the susceptibility of *x_i_*, respectively. The probability of a person getting infected is then given by *p*(Infected_*i*_) = 1 − exp(−*λ_i_* Δ*t*). To simulate external reseeding, we randomly select one person each day and infect him/her with pandemic influenza if he/she is susceptible.

For the length of each phase in the transmission model, we did not define a fixed duration. Rather, guided by the values reported in Germann *et al*. (2006), we divided the phases into a number of smaller substages, each lasting approximately 5–6 h. This increases the granularity of the transmission model, and allows us to better scale the infectiousness profile upon consumption of medicine. The smaller substages do not include the susceptible and recovered phases. Hence, given the reported mean lengths of the exposed (1.2 days), pre-symptomatic (0.7 days) and infectious phase (4.1 days), in the model we divided them into 5, 3 and 17 substages, respectively. Simulating the model 20 000 times without any forms of intervention policies or diagnostic approaches gave us an average of 29 h for the exposed phase, 17 h for the pre-symptomatic phase, and 98.5 h for the infectious phase. Assuming that the total duration of infection follows a normal distribution, we computed the mean length to be 144.5 h and a standard deviation of 6.13 h.

Following previous works, presumably 33 per cent of the infected people are asymptomatic (i.e. do not show any symptoms) while the remaining 67 per cent show symptoms of infection (Longini *et al*. 2005; Germann *et al*. 2006; Wu *et al*. 2006). Of those who are symptomatic, 6 per cent are assumed critical and require hospitalization (Wu *et al*. 2006). The other 94 per cent will show only mild symptoms. For the critically ill, they are 50 per cent more infectious as compared with those showing mild symptoms, while the asymptomatic cases are 50 per cent less infectious. In the current model, we ignored the occurrence of deaths. However, it can be assumed that the death rate is directly proportional to the total attack rate. For each individual, the susceptibility is assumed to be constant (unless the person is on medication, or immunized against the disease). However, the infectiousness (for a mildly symptomatic person) follows a baseline profile as shown in figure 1*b*. Both the susceptibility and infectiousness can be modified by the consumption of antiviral drugs. While an infected person is on medication, his/her infectiousness is reduced by 60 per cent. On the other hand, the susceptibility of an uninfected person will be reduced by 30 per cent. In addition, the probability of symptoms appearing will be reduced to a minimum of 23.45 per cent depending on the length of medication. Being on medication also shortens the duration of the disease by a maximum of 25 h if the consumption of drugs is sustained throughout the course of infection. Finally, once a person has recovered from the disease, he/she is assumed to be immune to further infection, and we set the susceptibility to 0 (see the electronic supplementary material for more details).

### 2.3. Handling index cases

**Figure 2.**
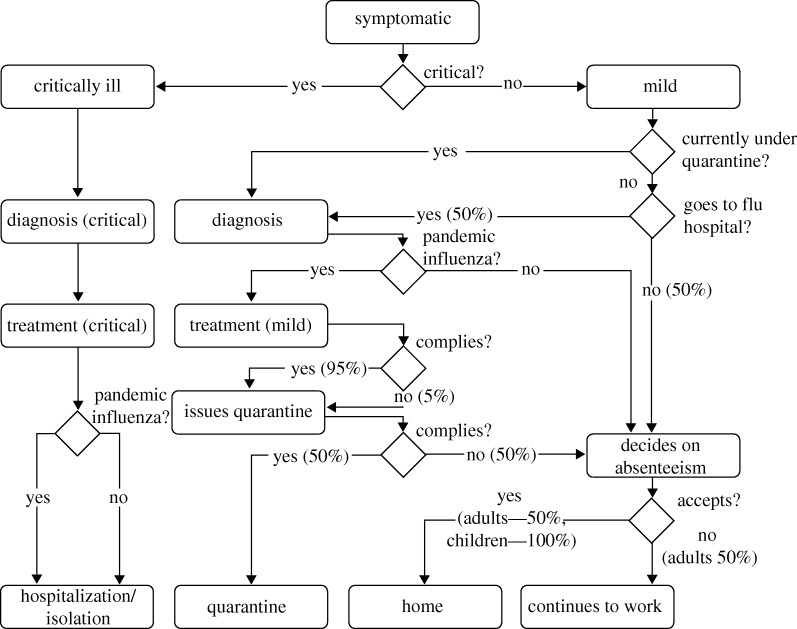
Decision flowchart (index cases). The decision flowchart showing the steps for handling index cases. The probability for making any decision is associated with the edges.

In our model, each index case is handled by a decision analytical model. The overview of the decision flowchart is described in figure 2. For simplicity, it is also assumed that each decision is made independently of other index cases. For instance, in the event that two or more people in the same household display flu-like symptoms, the decision for each of them visiting a doctor is made independently. The probability of each decision is also independent of any previous decisions made. To simulate scenarios where some intervention policies are not put in place, the associated probabilities are simply adjusted to reflect them accordingly.

The decision process is triggered when a person starts to show symptoms. Depending on the severity, if the person is critically ill, he/she is admitted to the flu hospital for diagnosis and given mandatory treatment. Regardless of diagnosis outcome, the person will be hospitalized and kept isolated. For one showing mild symptoms, there is a 50 per cent chance that he/she will visit the hospital to seek medical attention, i.e. diagnosis compliance rate. Note that the diagnosis compliance rate is 100 per cent for a person who is currently following quarantine orders. As our model is simulated in steps of 1 h, it will not be the case whereby the person will visit the hospital (if he/she decides to go) the moment symptoms appear. Instead, the trip will only be made after a certain delay, or when the hospital is open, whichever comes later. Currently, we set the delay to 6 h.

At the hospital, a patient is diagnosed using one of the four possible approaches, depending on the diagnostic test we wished to assess. The time required varies with the type of diagnostic tests being used. If the patient is tested positive for pandemic influenza, treatment will be offered. For the treatment, we assumed that the drug used is the commonly stockpiled Oseltamivir (US National Library of Medicine and National Institutes of Health 2009). A patient being tested positive (index case) will be given two doses a day for 5 days, consuming a total of 10 doses. A negatively tested patient will be sent home where he/she will decide whether or not to take absenteeism from work or school. Unlike patients who are admitted due to critical illnesses, the class of positively tested patients may not comply with the given treatment (i.e. refuse to take medication even when it is prescribed to them). In the current model, the treatment compliance rate is set at 95 per cent. However, regardless of compliance, drugs given are considered expended and will be added to the total medication used. After the required drugs have been prescribed, patients with mild symptoms will be issued quarantine orders, where they are to stay at home for a pre-determined amount of time. Again, the patient may or may not comply with the quarantine order. Currently, we set the quarantine compliance rate at 50 per cent.

A person with mild symptoms may refuse to go to the hospital, or he/she could be tested negative for pandemic flu, or simply does not comply with quarantine orders. Nonetheless, he/she is still unwell and may decide to take absenteeism from work (for adults) or school (for children). For our simulation, adults have a 50 per cent chance of absenting themselves from work, while children will always stay away from school, until they recover.

### 2.4. Contact tracing

Contact tracing is a non-pharmaceutical social distancing measure for limiting the spread of influenza by identifying people who may have come into contact with an infected person, and giving them prophylaxis or issuing them with quarantine orders. It has been shown theoretically that through contact tracing, major outbreaks can be reduced significantly at a small additional cost (Huerta & Tsimring 2002). We divided the contacts of each patient into one of the following three categories: household, workplace and friends. When contact tracing for a class has been activated, i.e. the index case is being tested positive for pandemic influenza, all the contacts in that class will be traced successfully. To emulate logistical delays in tracing them, we set the time for successful traces to be uniformly distributed between 5 and 7 h after positive diagnosis of the index case.

**Figure 3.**
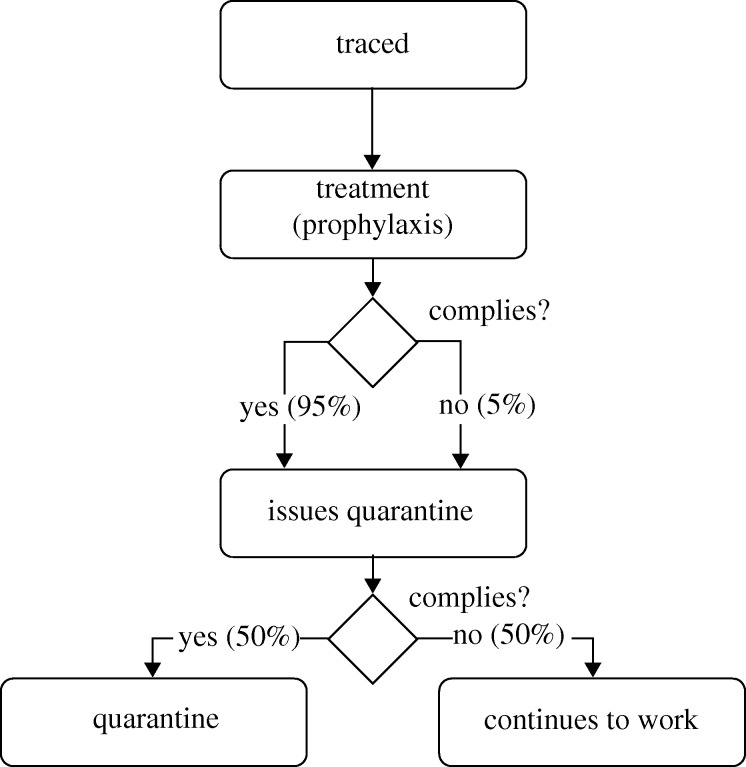
Decision flowchart (contacts). The decision flowchart for handling traced contacts for a typical intervention policy. For policies where certain steps are not being implemented, e.g. quarantine, the compliance probability is simply reduced to 0%.

Similar to the index cases, contacts are handled according to a decision flowchart as shown in figure 3. Upon being traced, the person will be offered prophylaxis—giving a person who is not obviously sick a course of antiviral drugs, so as to prevent him/her from being infected, or to reduce the severity of the illness when he/she does get infected. Again, there is a 95 per cent chance of the person consuming the medication. The prescription to be given is 1 dosage per day, for 10 days.

An insufficient drug stockpile is a real world issue faced by all countries. For instance, the United States has only enough antiviral medicine for 25 per cent of its population (US Department of Health & Human Services 2005). In order to conserve limited drug supplies, different countries have implemented policies that prioritize intended drug recipients according to their risk groups (US Department of Health & Human Services 2005). In addition, prophylaxis is only administered if deemed necessary. However, Longini *et al*. (2004) demonstrated that targeted antiviral prophylaxis could be an effective measure for containing influenza until the proper vaccines are developed. Here, some of the intervention policies include prophylaxis being given to contacts. In addition, to prevent drug wastage, we tracked the actual amount of drugs being consumed by the contact. If the contact becomes symptomatic later on and is subsequently diagnosed with pandemic flu, he/she will only be issued enough drugs to top-up his/her current supply to the required dosage levels as per index cases. Taking into account that not all patients will be upfront about the amount of drugs they already have, we set the average declared amount to be 50 per cent of the actual dosages remaining.

### 2.5. Intervention policies and diagnostic testkits

The various intervention policies implemented in our model consist of different combinations of handling index cases and their contacts. Specifically, we have the following intervention policies:

— P1: base case (no controls),
— P2a: treatment of index cases only,
— P2b: treatment and quarantine index cases,
— P3a: as per P2b, trace household contacts only, with 10 days prophylaxis,
— P3b: as per P2b, trace household contacts only, with 2 days quarantine,
— P3c: as per P2b, trace household contacts only, with both prophylaxis and quarantine,
— P4a: as per P2b, trace all contacts, with 10 days prophylaxis,
— P4b: as per P2b, trace all contacts, with 2 days quarantine, and
— P4c: as per P2b, trace all contacts, with both prophylaxis and quarantine.

Note that the duration of quarantine is set at 2 days as it is the length of time after exposure for symptoms to appear. For strains where the incubation period is longer, the quarantine duration may need to be adjusted accordingly. With the exception of P1, each intervention policy will be used with a particular testing method for the diagnosis of individuals who show influenza-like symptoms. The diagnostic approaches are:

— D1: assume all symptomatic cases are pandemic influenza positive,
— D2: fast testkit,
— D3: slow testkit, but the patient will resume his/her schedule during the testing period, and
— D4: slow testkit, with the patient staying in the hospital while waiting.

For the base diagnosis approach (D1), we assumed that all the people who display influenza-like symptoms are pandemic positive (regardless of whether they are really infected with pandemic virus) and are handled according to the prevailing intervention policies. Through this approach, there is no need for any testkits.

If fast testkits are to be used (D2), a patient will only have to spend 1 h in the hospital for diagnosis and the result (of whether he/she has contracted pandemic influenza) to be released. Alternatively, slow testkits can be used. However, with slow testkits, there is a waiting time of 12 h. Depending on the diagnostic approach, the patient can either resume his/her daily routine (D3), or wait in the hospital (D4). Under D3, if the test turns out positive the person will be recalled back to the hospital where the appropriate treatment (for policies P2–P4 only) is carried out. We assumed that the recall success rate is 100 per cent. By default, all tests (regardless of actual techniques) are set to be 100 per cent specific and 70 per cent sensitive.

## 3. Results

For each intervention policy and diagnostic approach, we ran the model 50 times, with each iteration simulating the spread of the influenza virus for 60 days. We assumed that a global pandemic is underway, and the city is already in the mitigation phase of its pandemic response strategy, whereby the aim is to reduce the total number of people affected and maximize care for those infected. We examined the efficiency of the various testkits in mitigating the outbreak within the city when positive cases have already appeared.

We measured the severity of the pandemic (and hence the efficacy of the diagnostic method) using two key indicators—total attack rate (AR_Total_) and peak attack rate (AR_Peak_). The total attack rate indicates the total number of people who display influenza-like symptoms due to the novel strain. It is not the same as the total number of people infected as there is a fraction of people who recover without showing any symptoms. The peak attack rate measures the highest number of people (at any one time point) showing symptoms due to the pandemic virus infection. It is an indication of the maximum burden on the healthcare system, as well as a partial reflection of the economic impact due to absenteeism among working adults.

Other auxiliary indicators measured are the costs incurred due to the amount of testkits being used (*C*_t_) and the antiviral drugs being issued (*C*_d_). This is in view of the limited supply of drugs and additionally, in our case, diagnostic testkits. Another resource that is often overlooked is the holding capacity of the influenza hospital. While patients are waiting for diagnosis or their test results, they are required to stay at the hospital (with the exception of diagnostic approach D3). Hence the peak waiting room occupancy (*C*_w_)—the maximum number of people waiting at any one time—is another indicator that is measured. To gauge the economic impact of the various intervention policies, we measure the number of work hours lost (*C*_h_) by all individuals who underwent diagnosis, treatment or were traced.

We formulated a function to compute the aggregate cost (*C*_Aggr_) incurred by the various intervention policies and diagnostic methods. This function is described by the following equation:
3.1
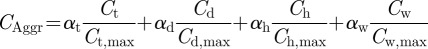

such that
3.2
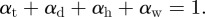

*C*_Aggr_ is a value that ranges from 0 to 1. The term *α* is the weight associated with the type of resource and it reflects the importance of each component to the aggregate cost. Note that there are no universal values for the weights, and they vary across cities with different resource priority. For instance, a model depicting a developed nation may be assigned a higher value for *α*_h_ due to its requirement to remain economically competitive whereas for a country without a proper healthcare system, one may assign higher values for *α*_d_ and *α*_t_ instead. Currently, we set *α*_t_ = 0.15, *α*_d_ = 0.45, *α*_h_ = 0.35 and *α*_w_ = 0.05 as we are investigating the use of testkits in a small city of a developed nation, but at the same time, we wish to minimize drug wastage.

### 3.1. Simulation with default parameters

**Figure 4.**
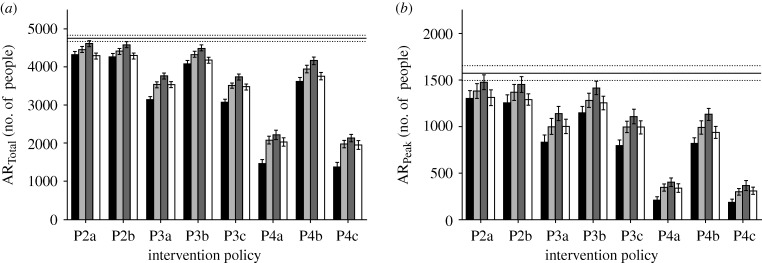
Total and peak attack rates. The diagram shows (*a*) the total attack rate and (*b*) peak attack rate for the various intervention policies. Policy P1 is shown as the base case as it depicts the case where there is no intervention (black bar, D1; light grey bar, D2; dark grey bar, D3; white bar, D4; solid line, base P1).

**Figure 5.**
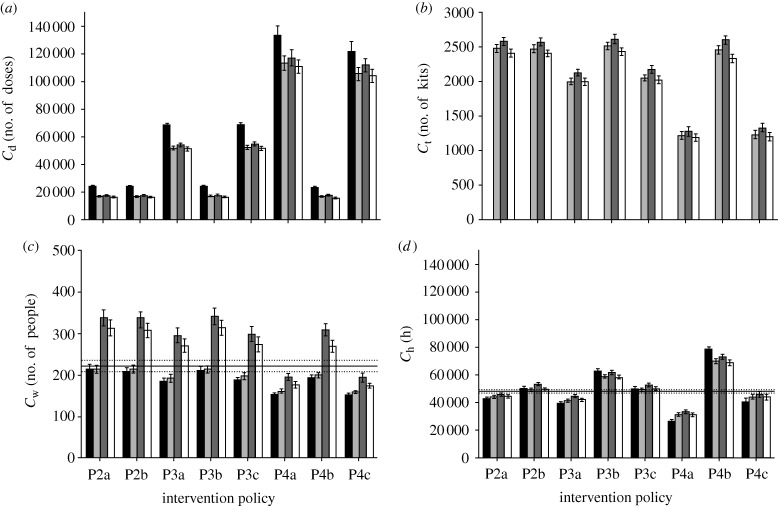
Antiviral drug usage, testkit usage and peak waiting room occupancy. The diagram shows the (*a*) drug usage, (*b*) testkit usage, (*c*) peak waiting room occupancy and (*d*) total work hours lost for the intervention policies. For (*a*,*b*), the base line is 0 as there is neither intervention nor treatment given to patients. For the usage of testkits (*b*), diagnostic approach D1 shows 0 usage since it is assumed all patients that show symptoms are infected with pandemic influenza (black bar, D1; light grey bar, D2; dark grey bar, D3; white bar, D4; solid line, base P1).

We first simulated the spread of the pandemic influenza under various intervention policies and diagnostic methods using the default parameters (see the electronic supplementary material). For the testkits, we assumed a specificity of 100 per cent and sensitivity of 70 per cent. From our simulations, the basic reproduction number (*R*_0_) is computed to be approximately 1.9, representing a moderately infectious pandemic outbreak. The results are shown in figures 4 and 5.

There is generally a similar trend in the comparison of the total attack rates, the peak attack rates and the amount of testkits being used across the different intervention policies, regardless of the diagnostic approach. An increase in the total attack rate corresponds to an increase in the peak attack rate and amount of diagnostic testkits being used. At the same time, to mitigate pandemic influenza, i.e. reduce the total attack rate, more antiviral drugs need to be consumed, a policy that may not be always feasible due to limited supplies. When treating only the index cases (figure 4*a*, policies P2a and P2b), the total attack rate shows a moderate decrease, from an average of 4463.9 cases (base line) to the lowest rate of 4016.2 cases (figure 4*a*, policy P2b, diagnosis approach D1). We start to observe a more significant decrease only when contact tracing comes into effect. Even so, this decrease is only significant when prophylaxis of the contacts is included in the policies.

The work hours lost from the various diagnostic approaches and intervention policies, interestingly, does not follow the same general patterns observed in the other indicators. In particular, increasing the coverage of contact tracing and assuming all cases to be pandemic does not necessarily equate to more work hours being lost (figure 5*d*, policies P4a and P4c, diagnosis D1). This is due to the decrease in total attack rate, which offsets the work hours lost by the contacts.

Within each implemented intervention policy, the impact of having a fast testkit on the total and peak attack rates is only matched when a slower diagnosis method is used and the patient stays at the hospital. In some cases, requiring the patients to stay while waiting for the test results may even be more effective in containing the virus, as exemplified by policies P3b and P4b shown in figure 4*a*. However, the peak waiting time associated with such an approach may prohibit its effective implementation. Of course, one can simply assume that all patients who exhibit influenza-like symptoms have contracted pandemic influenza and issue them with the antiviral drugs. The downside of this approach is then the increased drug usage, and the proportion being wasted due to consumption by those inflicted with ILIs (figure 5*a*). Nonetheless, it may still be a viable option if the testkits constitute another limited resource that has to be managed properly.

**Figure 6.**
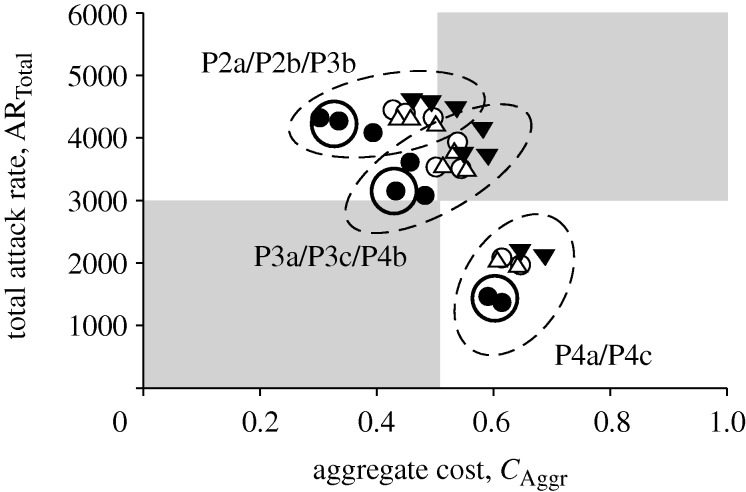
Total attack rate against aggregate cost. Each point in the scatter plot corresponds to an intervention policy and diagnostic method. Points of the same shape refer to the same diagnosis method but with different intervention strategies. The plot area can be divided into four quadrants, with the lower-left depicting the most optimal outcome. Data points can be grouped into clusters, shown by dashed circles. The points in each cluster are derived from one of the stated intervention policies (filled circle, D1; open circle, D2; inverted triangle, D3; open triangle, D4).

Figure 6 shows the plot comparing the total attack rate with the aggregate cost. From the plot, we see that in the context of most intervention policies, diagnostic approach D1 is distributed closer to the desired lower-left quadrant—which corresponds to an optimal ratio of number of people infected compared with resources consumed—especially when contact tracing has been implemented. Even with the use of diagnostic testkits, a slower one that requires the patient to stay, coupled with an appropriate intervention policy, is comparable to using a fast testkit. The only consideration for such an approach would then be the peak hospital waiting room occupancy. However, given that the availabilities of antiviral drugs and testkits are usually the main limiting factors, a slow testkit with the patient waiting in the hospital may still be preferred when a fast and accurate kit is not yet ready.

In addition, using the current weights for the aggregate cost function, data points derived from the same intervention policies can be clustered and grouped together (shown in figure 6 as dashed circles). This suggests that, based on the current priorities given to the cost components, intervention policies are the dominating factors in mitigating an influenza pandemic. It should be noted that, in particular, prophylaxis policies guided by contact tracing have stronger effects on reducing the total attack rate (lower right quadrant in figure 6). Within each intervention policy, the different diagnostic methods then fine-tune the allocation of limited resources.

### 3.2. Sensitivity analysis

#### 3.2.1. Basic reproduction number.

The basic reproduction number *R*_0_ is an indicator that is often used to measure the transmissibility of a pandemic outbreak. Simply put, it is the average number of other individuals each infected person will infect in a completely susceptible population. Analyses of previous influenza pandemics estimated *R*_0_ for large communities to lie between 1.2 and 3 (Mills *et al*. 2004; White & Pagano 2008). For the recent H1N1 pandemic, some early studies have suggested *R*_0_ to range between 1.4 and 1.6 (Fraser *et al*. 2009)—indicating relatively low transmissibility—while others estimated it to be as high as 3.1 in certain countries such as Mexico (Boёlle *et al*. 2009).

We obtained *R*_0_ by first turning off all intervention policies prior to simulation. We then randomly infect one susceptible person and simulate the model, noting down the number of people he/she transmits the virus to. This process is repeated 1000 times, and the average number of transmissions is reported as the *R*_0_ value. With the default parameters, we computed *R*_0_ in the model to be approximately 1.9. To see the effectiveness of the various diagnostic approaches and intervention policies under different viral transmissibility, we vary *R*_0_ from 1.6 (low transmissibility) to 2.3 (high transmissibility) by manually adjusting the transmission coefficients *β*_h_, *β*_w_ and *β*_c_ (equation (2.1)), while ensuring that the transmission proportion between the different location types remains relatively constant.

**Figure 7.**
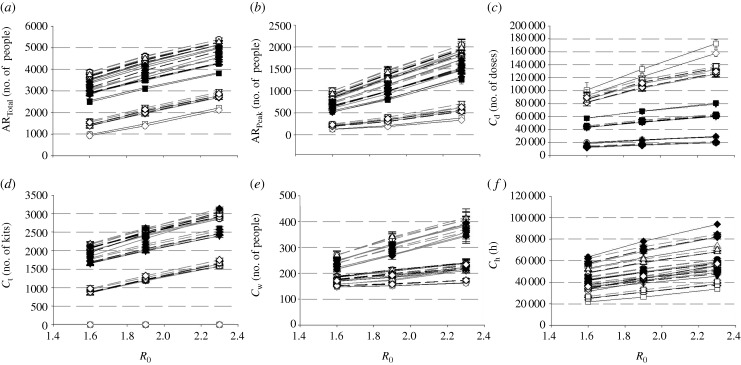
Varying the *R*_0_ values. The graphs show how each indicator changes with increasing *R*_0_ values. Data points obtained from the different intervention policies are identified by unique symbols, while the connecting lines are used to differentiate between the diagnostic approaches. All the indicators increase in an almost linear fashion with higher *R*_0_ values. In addition, data points from the same intervention policies tend to cluster together, as shown by the general proximity of similarly shaped symbols. Hence, it is the policies that largely dominate the outcomes of influenza mitigation, regardless of transmissibility. (*a*) Total attack rate; (*b*) peak attack rate; (*c*) antiviral drug usage; (*d*) testkit usage; (*e*) peak waiting room occupancy; (*f*) work hours lost (filled circle, P2a; open circle, P2b; filled inverted triangle, P3a; open inverted triangle, P3b; filled square, P3c; open square, P4a; filled diamond, P4b; open diamond, P4c; solid line, D1; dark-grey dashed line, D2; light-grey dashed line, D3; light grey solid line, D4).

Figure 7 shows the changes of various indicators (AR_Total_, AR_Peak_, *C*_d_, *C*_t_, *C*_w_ and *C*_h_) for different diagnostic methods and intervention policies with varying transmissibility. In general, there is a monotonic increase across all indicators as *R*_0_ increases. Again, within each graph, it is observed that values from the same intervention policy tend to cluster together, regardless of viral transmissibility (with the exception of testkit usage for diagnostic approach D1, figure 7*d*). Hence during the onset of an outbreak, where the transmissibility is still unknown—which is often the case—it may be more prudent to first implement proper social distancing intervention policies, followed by the use of testkits to ensure that drug wastage is minimized.

#### 3.2.2. Compliance rate.

In the model, the current compliance rates for diagnosis, quarantine and absenteeism are set at 50 per cent (figure 2). However, in the event of a pandemic outbreak, there may be heightened awareness among the population, such that individuals displaying flu-like symptoms are now more likely to seek medical attention and follow quarantine orders. To investigate the effects of varying compliance, we simulate the model with varying levels of compliance rates (from 30 to 70% for a medium transmissible pandemic outbreak, i.e. *R*_0_ = 1.9).

**Figure 8.**
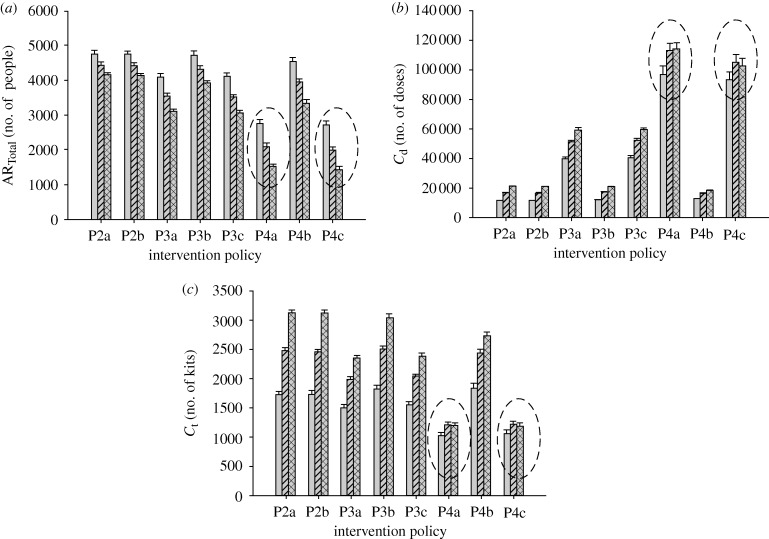
Effects of varying compliance rates. The graphs show the changes of the (*a*) total attack rate, (*b*) drug usage and (*c*) testkit usage with increasing compliance rates for a particular diagnostic approach (D2). In particular, for policies P4a and P4c (highlighted in dashed circles), higher compliance rate (from 50 to 70%) will result in lower total attack rate, but without a corresponding increase in the resources required (light grey bar, 30% compliance; striped bar, 50% compliance; checked bar, 70% compliance).

The graphs in figure 8 show the results of varying the compliance rates. The general trend shows total attack rate decreasing as more symptomatic individuals seek some forms of diagnosis or antiviral medication. One would expect that with more people visiting the hospital, the amount of drug and testkit usage will increase as well. However, the contrary is observed for some cases, particularly for policies P4a and P4c. Under these policies, the total attack rate can drop by as much as 28.4 per cent as the compliance rates increase from 50 to 70 per cent (policy P4c, diagnosis approach D2), and instead of seeing an increase in the medicine consumption, we get a decrease by 2.3 per cent (figure 8). Similar patterns are observed when other diagnostic approaches are implemented in tandem with P4a and P4c. We noted that the reversal of the correlation between total attack rate and cost occurs only in the presence of two conditions: (i) full contact tracing and (ii) prophylaxis of the contacts. Additionally, a high diagnosis compliance rate can be loosely associated with a lower false negative rate for viral detection. Hence, we expect measures that reduce the false negative rate to have a more significant impact on pandemic mitigation when implemented with the abovementioned conditions. The critical issue that follows is then how limited resources such as antiviral drugs and testkits can be used effectively with those conditions.

#### 3.2.3. Antiviral drug efficacy.

The effectiveness of antiviral drugs may be a potential factor influencing the impact of intervention policies and testkits on pandemic outbreak mitigation. Drug efficacy is represented in the model by the levels of reduction to infectiousness and susceptibility, as well as the probability of symptoms appearing for an infected person. We simulated the scenario for lower drug efficacy by lowering the amount of infectiousness reduction from 60 to 30 per cent, and susceptibility reduction from 30 to 15 per cent for infected and uninfected individuals, respectively. In addition, there is also a reduction of symptomatic probability to 45 per cent.

**Figure 9.**
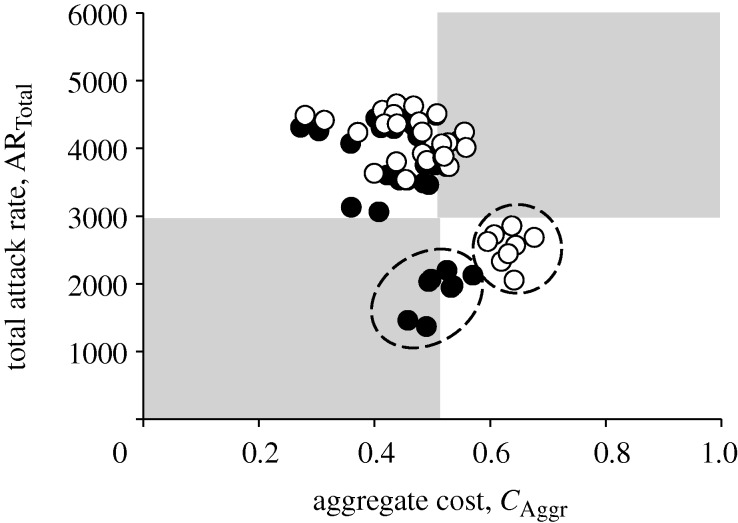
Reduced drug efficacy. The black dots are the various data points obtained by simulating the model using base drug efficacy (base data points), while the white ones are derived using reduced drug efficacy (reduced data points). The aggregate cost for the base data points differs slightly from the original plot (figure 6) due to the increased set of data points used in the computation. Most of the reduced data points are shifted by a small amount, except for those obtained using policies P4a and P4c, as indicated by dashed circles.

To examine the effects of lower drug efficacy, we look at the scatter plot of total attack rate against aggregate cost for both sets of simulations—base and reduced drug efficacy (figure 9). From the plot, there is a general increase in both total attack rates and costs with decreased drug effectiveness. Again, the results can be clustered by the intervention policies. However, it is interesting to note that, similar to varying the diagnosis compliance rate, points from policies P4a and P4c collectively experience a larger amount shift along both axes. Conversely, for a more effective antiviral drug, the impact on reducing both attack rates and costs through the implementation of policies involving full contact tracing and prophylaxis to the contacts will most probably be more significant.

#### 3.2.4. Testkit sensitivity.

**Figure 10.**
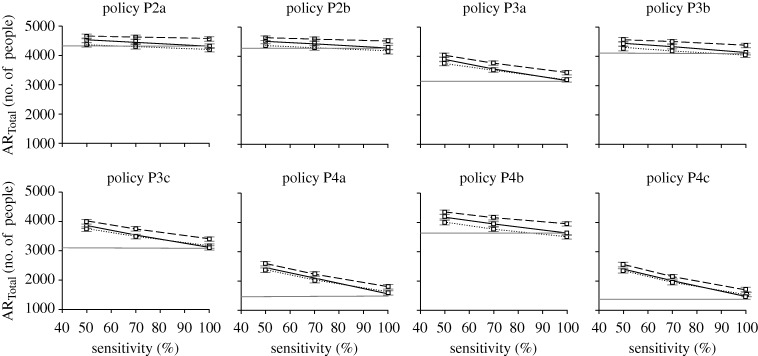
Testkit sensitivity on total attack rate. Graphs showing how testkit sensitivity affects the total attack rate for the different intervention policies and diagnostic approaches. The horizontal straight line indicates approach D1, where all symptomatic individuals are assumed to be positively infected with pandemic virus (solid grey line, D1; solid black line, D2; dashed line, D3; dotted line, D4).

To test the robustness of the observations for diagnostic approaches D2 to D4 within each intervention policy, the simulations are repeated but with varying levels of testkit sensitivity (50 and 100% sensitive). In general, increasing the sensitivity of the testkits has the effect of decreasing the total attack rate (figure 10). Surprisingly in some cases, a highly sensitive, but slow testkit, coupled with proper diagnostic approaches, can bring about a lower total attack rate as compared with simply assuming that all patients are infected with pandemic influenza (figure 10, diagnosis D4 with policies P2a, P2b, P3b and P4b). One possible explanation is that confining the pandemic-positive patients in the hospital tends to remove them from infectious circulation while they are at their peak infectiousness.

**Figure 11.**
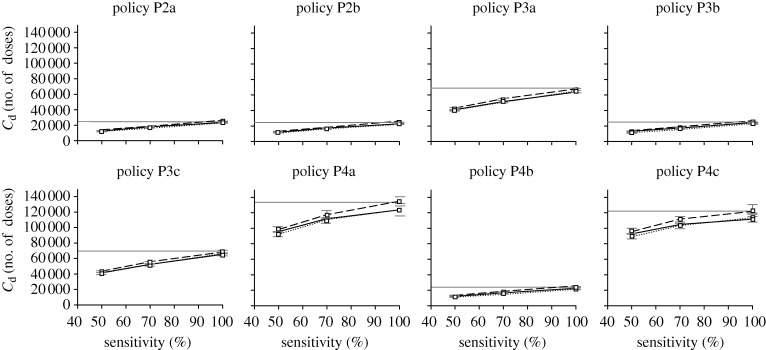
Testkit sensitivity on antiviral drug usage. Graphs showing how testkit sensitivity affects the amount of antiviral drug usage for the different intervention policies and diagnostic approaches (solid grey line, D1; solid black line, D2; dashed line, D3; dotted line, D4).

**Figure 12.**
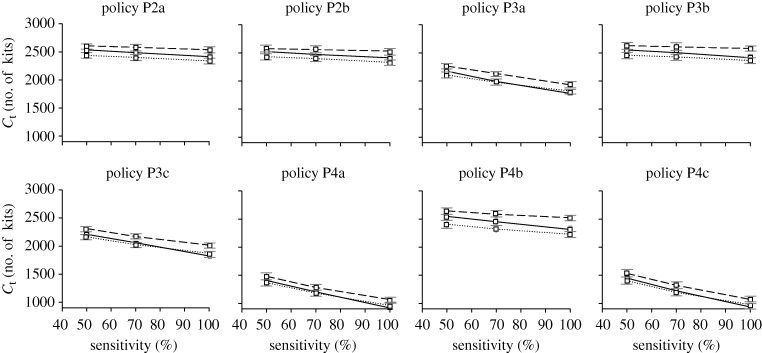
Testkit sensitivity on testkit usage. Graphs showing testkit usage with different testkit sensitivity. Note that D1 is not present in this graph as no testkits are being used using that approach (solid black line, D2; dashed line, D3; dotted line, D4).

Despite the decrease in total attack rate, the amount of drug usage increases with better sensitivity, mainly due to more people being correctly diagnosed and administered with antiviral drugs. This increase is not observed for the amount of testkits being used (figures 11 and 12). One interesting observation is that as the sensitivity of the testkits improves, the amount of drug usage becomes almost as much as if we were to assume that all patients are positively infected with pandemic influenza (figure 11). In some cases, the amount of drug dosage even exceeds the base approach D1 (e.g. figure 11, policy P4c, diagnosis D2), possibly due to the higher total attack rate. Regardless of the testkit sensitivity, variations to the amounts of drug and testkit usage between diagnostic approaches D2 to D4 are not as significant as compared with the variations due to the different intervention policies being implemented.

### 3.3. Hospital segregation

Hospitals provide another dimension in shaping the spread of an influenza pandemic and affecting the efficacy of the various policies and diagnostic approaches. Unlike other location types, individuals who show influenza-like symptoms will deliberately go to the hospital for diagnosis, providing an opportunity for potential spread of the virus (Nuño *et al*. 2007). In our current model setting, hospital transmission does not play a major role as we have implemented a simple hospital segregation policy through the designation of a flu hospital. However, without such policies, some diagnostic methods may not work as well as shown previously.

We briefly study the importance of implementing proper hospital segregation policies by simulating our model without a designated flu hospital. Each symptomatic person will randomly select a hospital to visit for diagnosis and treatment. In addition, we increase the number of ONILI cases by threefold to 135 individuals daily in order to assess the results under a more severe setting. These people will also go to any of the two hospitals for treatment with a 50 per cent compliance probability.

**Figure 13.**
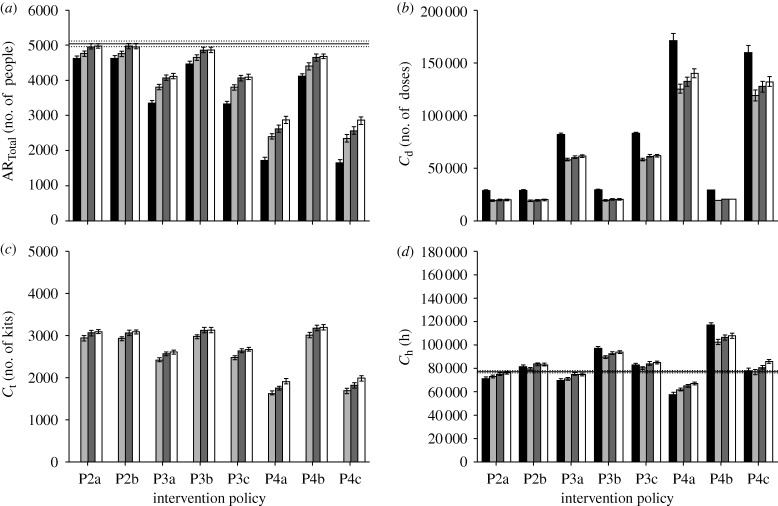
Simulation results without hospital segregation. The diagram shows the (*a*) total attack rate, (*b*) drug usage, (*c*) testkit usage and (*d*) total work hours lost for the various intervention policies and diagnostic approaches without designating one hospital as a flu hospital. Note that for all cases, D4 has a higher total attack rate and it consumes more resources as compared with D2 and D3 (black bar, D1; light grey bar, D2; dark grey bar, D3; white bar, D4; solid line, base P1).

The total attack rate, drug usage, testkit usage and amount of work hours lost are shown in figure 13. From the results, we see an overall increase in the total number of people infected and the consumption of the different resources. However, an interesting observation is that the effectiveness for diagnostic approach D4—where the patient stays in the hospital while waiting for the test results—starts to degrade regardless of intervention policy. One reason for this phenomenon is that the force of infection experienced by an ONILI patient is increased dramatically, not only because they visit the same hospital as infected individuals, but also due to the higher concentration of infectious people in the hospitals as compared with other location types. In fact, in the absence of a fast testkit, simulations showed that it is better to collect the samples from the patient and allow him/her to go off while diagnosis is being performed using conventional laboratory-based methods. Nonetheless, the better policy will still be to prevent the mixing of genuine influenza cases and those with ONILIs.

## 4. Discussion

From the simulation results presented in the previous section, the impact of a fast testkit on mitigating pandemic influenza may not be as significant as the various social distancing policies themselves. In particular, for most cases within our model set up, assuming that the patient is positive for pandemic flu will result in better allocation of limited resources (drug dosage and amount of testkits) while minimizing the total number of people being infected. Then why is a fast testkit necessary? In the current model, the implementations of diagnostic methods and intervention policies are homogeneous, i.e. assume pandemic positive, slow and fast testkits are not being used in a way such that their intended effects are maximized according to whether the individual is an index case or a contact. In general, we find that three of the most important factors that guide the use of diagnostic testkits are (i) the resource consumption by false positives, (ii) the frequency of false positives, and (iii) the propagation potential of false negatives.

In this work, an individual suspected to be infected may consume the following resources—antiviral drugs, testkits, work hours lost (which represents an intrinsic economic opportunity cost), and less importantly, the peak waiting room occupancy. Allocating an individual these resources when it is not really required (whether it is due to assuming that he/she is pandemic positive or the inadequate specificity of the testkits) is wastage which any policy maker would try to minimize. This is especially true for antiviral drugs, which often represents a hard constraint. Depending on the frequency and proportion of such false positives, proper diagnostic approaches can then be implemented. For instance, when the proportion of false positives is high, such as the case of a high ILI rate, assuming that all patients are pandemic positive would put a strain on the limited drug supplies. Of course, such rates may not be possible to obtain for a novel pandemic strain with no outbreak history. Nonetheless, by not advocating a blanket approach and instead, identifying areas where false positives are most likely to occur, the proper use of fast testkits can possibly achieve better mitigation outcomes and minimizing wastage.

To further illustrate our point, when a local pandemic is already underway, as depicted by our model, most patients who turn up at the hospitals for diagnosis are likely to have been inflicted with pandemic influenza. As such, it may be more prudent to assume positive and administer antiviral drugs without diagnosis. However, a different approach may be adopted for their contacts. From figure 5*a*, there is a significant increase in the amount of drugs being used when the tracing of all contacts is in place (except for policy P4b). Policy P4a uses almost 5 times more drugs than policy P2a despite reducing the total attack rate by only slightly more than half. This suggests that there is a huge amount of wastage due to the contacts. Hence, instead of applying prophylaxis to the contacts, using diagnostic testkits to identify infected contacts and treating them with antiviral drugs might then possibly yield a better attack rate-to-drug usage ratio. The question then remains as to whether a fast or slow testkit makes a difference to the attack rate and drug usage. Assuming that the contacts need not go to the hospital and instead a respiratory specimen is collected and sent for analysis while the contact is ordered to stay at home (a diagnostic approach similar to D4), then a fast testkit may not be required. However, if a trip to the hospital is needed for diagnosis, the availability of a fast testkit may minimize the time the contact spends not under quarantine, preventing further spread of the influenza virus.

Another factor influencing the necessity of testkits, which is closely related to their sensitivity, is the repercussions of not treating a pandemic-positive individual. In figure 4, the total attack and peak attack rates for non-treatment are shown as base lines. For a moderately infectious variant as simulated in our model, an untreated case may not spread the virus in an exponential manner. As such, a testkit may be used to reduce drug wastage, but a highly sensitive one may not be required since the infectiousness of the individual is limited. However, for the more transmissible cases, such as the ongoing H1N1 swine flu outbreak (at the time of writing; Neumann *et al*. 2009; Novel Swine-Origin Influenza A (H1N1) Virus Investigation Team 2009), it may be unwise to leave a potentially infectious person untreated. If a sensitive testkit is not yet available, then assuming that a symptomatic individual has contracted the pandemic virus is probably more efficient in bringing down the attack rates. Aside from the transmissibility of the virus, geographical features may also affect the propagation potential of a false negative. In urban areas such as cities, where close contact due to work and public transport is inevitable, a single untreated case can rapidly lead to the formation and spread of influenza clusters.

One other aspect which can be further investigated is the role of public transport in transmitting the pandemic virus. Public transport such as buses and subways not only facilitates the displacement of carriers between locations within the city, it also brings several individuals into close proximity, increasing the likelihood of the virus infecting several other people. A limitation of current diagnostic measures in most countries is that a symptomatic person has to travel to a local hospital for testing, which requires commuting, mostly on public transport. As mentioned previously, it is possible for a symptomatic individual to either send a respiratory specimen to the hospital, or request for a fast testkit for diagnosis at home, hence alleviating the need for public transport. Both measures can potentially reduce the risk of exposure to others significantly.

There are several other aspects to pandemic influenza that determine the efficacy and necessity of a fast testkit in minimizing drug wastage while mitigating an outbreak. Parameters and policies such as compliance rate of the general public, as well as activating prophylaxis to priority groups (US Department of Health & Human Services 2005) will influence how a testkit can be effectively used. However, considering these parameters and policies is beyond the current scope of this work.

For future works, we may add more flexibility to the policies being implemented, such as differential handling of index cases and contacts as briefly discussed. One mitigation means that can also be further explored is the role of vaccines and how they would shape the spread of the pandemic outbreak. Additionally, more complex social interaction patterns (Barrett *et al*. 2008) can be implemented to represent the spread of the pandemic in a more realistic manner. Data mining techniques (Baily-Kellogg *et al*. 2006) can then be used on the simulation results to better assist policy makers in assessing the implications and effectiveness of their policies both spatially and temporally. Finally, the aggregate cost function (equation (3.1)) may also be modified to present a more systematic cost–utility assessment of the various diagnostic approaches and intervention policies, taking into account factors such as quality-adjusted life-years (Sander *et al*. 2009).

## 5. Conclusion

Managing limited resources such as testkits and antiviral drugs while trying to contain the spread of pandemic influenza is a major challenge faced by several countries. In the event where a fast and accurate testkit is not yet available, one has to rely on slower testkits, coupling their use with effective social distancing measures to minimize the wastage of antiviral drugs. In this work, we have developed a stochastic agent-based pandemic model to assess the necessity of a fast testkit. From our simulation results, we showed that intervention policies, and not testkits, are the key means to successfully contain an outbreak, and that casting a wider net for contact tracing is crucial for minimizing the total attack rate. However, although not yet included in our model, we can infer from the results that most of the drug wastage is due to prophylaxis being administered to the contacts. Considering the use of testkits on the contacts may be a better resource allocation strategy. Within each intervention policy, the use of slower testkits while holding the individual at the hospital can be an equally effective means of mitigating a pandemic outbreak as compared with a fast testkit. However, this is provided that proper infection controls, such as a hospital segregation policy, are in place. Otherwise, hospital transmission may limit the usefulness of other efforts such as contact tracing and quarantine orders.
